# Mechanical and Thermal Conductivity Properties of Enhanced Phases in Mg-Zn-Zr System from First Principles

**DOI:** 10.3390/ma11102010

**Published:** 2018-10-17

**Authors:** Shuo Wang, Yuhong Zhao, Huijun Guo, Feifei Lan, Hua Hou

**Affiliations:** School of Materials Science and Engineering, North University of China, Taiyuan 030051, China; wangshuo313@sina.cn (S.W.); ghjwwd@163.com (H.G.); lan.feifei@foxmail.com (F.L.); houhua@nuc.edu.cn (H.H.)

**Keywords:** Mg-Zn-Zr alloys, mechanical properties, thermal conductivity, density functional theory

## Abstract

In this paper, the mechanical properties and minimum thermal conductivity of ZnZr, Zn_2_Zr, Zn_2_Zr_3_, and MgZn_2_ are calculated from first principles. The results show that the considered Zn-Zr intermetallic compounds are effective strengthening phases compared to MgZn_2_ based on the calculated elastic constants and polycrystalline bulk modulus *B*, shear modulus *G*, and Young’s modulus *E*. Meanwhile, the strong Zn-Zr ionic bondings in ZnZr, Zn_2_Zr, and Zn_2_Zr_3_ alloys lead to the characteristics of a higher modulus but lower ductility than the MgZn_2_ alloy. The minimum thermal conductivity of ZnZr, Zn_2_Zr, Zn_2_Zr_3_, and MgZn_2_ is 0.48, 0.67, 0.68, and 0.49 W m^−1^ K^−1^, respectively, indicating that the thermal conductivity of the Mg-Zn-Zr alloy could be improved as the precipitation of Zn atoms from the α-Mg matrix to form the considered Zn-Zr binary alloys. Based on the analysis of the directional dependence of the minimum thermal conductivity, the minimum thermal conductivity in the direction of [110] can be identified as a crucial short limit for the considered Zn-Zr intermetallic compounds in Mg-Zn-Zr alloys.

## 1. Introduction

Grain refinement is a metallurgical phenomenon that has been exploited in magnesium alloys to achieve desired microstructure and mechanical properties. Zirconium, a powerful grain refiner, has been widely used in magnesium alloys [1,2,3]. Magnesium-zinc-zirconium (ZK) alloys mainly refer to those containing zirconium or grain refined by zirconium, such as ZE41, ZK60, WE43, ML12, and ML10, as well as OS-1–3, and such like, and these commercial Mg-Zn-Zr alloys comprise the basis of the current magnesium alloy business. It was previously agreed that grain refinement of magnesium alloys by Zr was noticeable at low levels of soluble Zr [4], but the subsequently detailed examinations showed that both insoluble zirconium particles and zirconium dissolved in the melt played a role in grain refinement [5]. These conditions require that the magnesium alloys contain maximum soluble and undissolved Zr content. Meanwhile, a large amount of Zr content could lead to the formation of Zn-Zr intermetallic compounds in Mg-Zn-Zr alloys.

Based on the study of the composition and distribution of zirconium, Morozova and Mukhina [6] proposed that the highly dispersed particles of Zn_2_Zr_3_, ZnZr, and Zn_2_Zr intermetallic compounds were the determining factors for the nano-structural mechanism of strengthening in the Mg-Zn-Zr system. Li et al. [7] proposed that the Mg-5Zn-2Gd-0.4Zr alloy (wt.%) showed a significant hardening response during aging, thus forming different morphologies, including ZnZr and Zn_2_Zr phases. Although the ZnZr_2_ and Zn_2_Zr_3_ phases do not belong to the ground state of the Zn-Zr system, they were dynamically stable at 0 K using the harmonic approximation [8]. Recently, it was reported that Mg-Zn-Zr alloys were also prospective to be ideal thermal conductive material for application in LED light fixtures [1,9]. With the aging process, Li et al. [10] suggested that the thermal conductivity of Mg-2Zn-Zr alloy obviously increased due to the precipitation of Zn atoms from the α-Mg matrix, accompanied by the formation of Zn-Zr precipitations, such as ZnZr, Zn_2_Zr, and Zn_2_Zr_3_. Similarly, Yamasaki and Kawamura [11] proposed the thermal conductivity of Mg-Zn-rare earth (RE) alloys exhibited higher thermal conductivity than their solution-treated counterparts due to the consumption of solute elements during the formation of the long-period stacking ordered phase (LPSO). Also, due to the formation of a rare-earth phase, the thermal conductivity of the Mg alloy was raised markedly with an increase in Sm content, which helped to dissolve the Zn atoms in the *α*-Mg matrix [12].

Laves MgZn_2_ is known to be the most important strengthening phase in Mg-Zn-Zr alloys. Due to the importance of the strengthening effects of the MgZn_2_ phase and the role of micro-alloying on precipitation strengthening, MgZn_2_ has been studied extensively through theoretical calculations to experimental exploration [13,14,15]. By adding grain-refining elements, the density of aging precipitate MgZn_2_ can be increased, thereby improving the mechanical properties of the alloy [15]. Meanwhile, it is also an effective way to dissolve the Zn atoms in the *α*-Mg matrix by forming the MgZn_2_ phase. However, to our knowledge, theoretical research regarding mechanical properties and thermal conductivities of Zn-Zr intermetallic compounds compared with MgZn_2_ are relatively scarce.

In addition, experimental information is quite limited in establishing phase/property relationships for these precipitations. Nevertheless, the first principles calculations may be an available approach to research these properties. Accordingly, the mechanical and thermal conductivity properties of ZnZr, Zn_2_Zr, Zn_2_Zr_3_, and MgZn_2_ have been conducted through the first-principles calculations. Generally, the minimum thermal conductivity can be used to identify candidate materials for high-temperature applications. In this contribution, the minimum thermal conductivity, according to the modified Clarke’s model [16] is investigated for intermetallic compounds ZnZr, Zn_2_Zr, and Zn_2_Zr_3_. In addition, the direction-dependent minimum thermal conductivity, based on the Cahill’s model [17] has been further studied in-depth to understand the effect of the Zn-Zr precipitations on the thermal conductivity properties of Mg-Zn-Zr alloys. Undoubtedly, it is anticipated that the results will guide people’s selections of the appropriate ZK alloys for different applications.

## 2. Computational Details

All calculations in this work were performed by using the Vienna ab initio simulation package code (VASP) [18] within the generalized gradient approximation (GGA) [19] of Perdew-Burke-Ernzerhof (PBE) [20] exchange correlation density functional. The electron configuration treated 3s^2^ as a valence state for Mg, 3d^10^4s^2^ as a valence state for Zn, and 4s^2^4p^6^5s^2^4d^2^ for Zr, respectively. Extensive convergence tests suggested that the cutoff energy of 400 eV was enough for all phases in the calculations. The special points sampling integration was used over the Brillouin zone with 7 × 7 × 3 and 8 × 8 × 8 k-points using the Gamma-centered Monkhorst-Pack method [21] for MgZn_2_ and the Zn-Zr system (including ZnZr, Zn_2_Zr and Zn_2_Zr_3_), respectively, in geometry optimization. The convergence criterion of the Hellmane Feynman force was 0.01 eV/Å for complete relaxation of the atomic positions within the maximum stress on the atom of 0.02 GPa. The electronic iterations convergence was 1.0 × 10^−5^ eV for the total energy calculated together with first-order Methfessel-Paxton smearing with a width of 0.2 eV. Considering the unfilled electron of the *4d* shell of the transition metal Zr, the spin polarization was considered in the calculation with the initial magnetic moment 3 *µ*_B_ according to Hund rules. 

For obtaining the equilibrium bulk modulus *B*_0_ of the spin state at 0 K, the ground state energy *E*_0_ as a function of the cell volume within the Birch-Murnaghan equation of states (EOS) [22] was applied. Meanwhile, in order to investigate chemical stability, the contribution of the lattice vibrations *F*_vib_ to the total Helmholtz free energy (*F = E*_0_ + *F*_vib_) was evaluated (it is worth noting that the contribution of the thermal electrons is negligible compared to lattice vibrations, and is therefore ignored in the current work). For the sake of computational efficiency, the vibrational free energy was derived by using the Debye-Grüneisen [23] model as follows:

(1) $$F_{\mathrm{vib}}\left(V,T\right)=\frac{9}{8}nk_{B}\Theta +T\left[3\ln\left(1-e^{-\Theta /T}\right)-D\left(\Theta /T\right)\right]$$

where *k*_B_ is the Boltzmann constant and *n* is the number of atoms per formula unit. The Debye temperature *Θ* was obtained as proposed by using [24]:

(2) $$\Theta_{D}=\frac{1}{K_{B}}{\left(6\pi^{2}V^{1/2}n\right)}^{1/3}f(\sigma )\sqrt{\frac{B_{0}}{M}}$$

where *M* is the molecular mass per primitive cell, and *B*_0_ and *σ* are the static bulk modulus and Poisson ratio at the equilibrium geometry, respectively. The *f*(σ) function is:

(3) $$f\left(\sigma \right)={\left\{3{\left[2{\left(\frac{2}{3}\frac{\left(1+\sigma \right)}{\left(1-2\sigma \right)}\right)}^{3/2}+{\left(\frac{1}{3}\frac{\left(1+\sigma \right)}{\left(1-\sigma \right)}\right)}^{3/2}\right]}^{-1}\right\}}^{1/3}$$

The elastic coefficients were determined by applying a set of given deformation with a finite value fitting the total energy of the crystal, as implemented by Mayer et al. [25]. In order to remain within the elastic limit of the selected phases, small strains up to ±2% at 0.5% interval were used.

## 3. Results and Discussion

### 3.1. Structure and Stability

**Figure 1:**
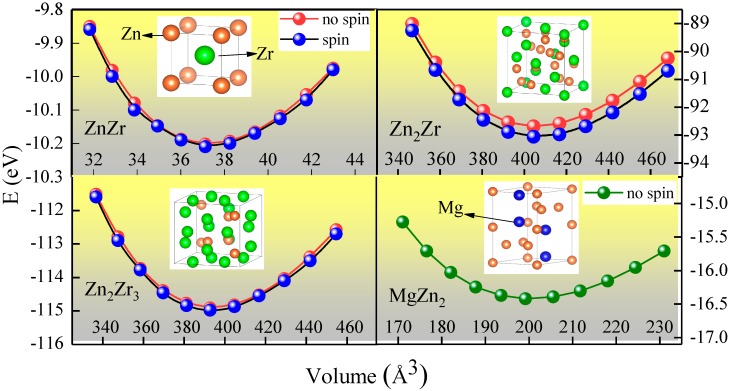
Total energy as a function of unit cell volume for ZnZr, Zn_2_Zr, and Zn_2_Zr_3_ at both no-spin and spin states, as well as MgZn_2_ at the nonmagnetic state.

Figure 1 shows the energy-volume fittings for ZnZr, Zn_2_Zr, Zn_2_Zr_3_ at both no-spin and spin states, as well as MgZn_2_ at a nonmagnetic state. It is clear that for energy that was dependent on the volume of the two states, the spin state was more energetically stable than the no-spin state for Zn-Zr intermetallic compounds, especially for the Zn_2_Zr phase. Thus, in the following discussion, we focus on properties at the magnetic state. The optimized lattice constants at the ground state and the corresponding fitting *B*_0_ for each Zn-Zr intermetallic compound are given in Table 1. As seen, the calculated lattice parameters of Zn-Zr intermetallic compounds and the MgZn_2_ phase are in good agreement with the available calculated results. When comparing the equilibrium bulk moduli of these four intermetallic compounds, which could describe the stiffness of the crystal to the applied strain, it can be observed that the *B*_0_ of the selected Zn-Zr compounds is interestingly larger than that of MgZn_2,_ which has generally been considered as the main strengthening phase in Mg-Zn alloys. We estimated the chemical stability based on the calculated Helmholtz free energy *F* (eV/atom) of Zn-Zr compounds. Generally speaking, these phases are thermodynamically stable due to negative Helmholtz free energy, which is considered to be a key factor for the alloys’ synthesis and stabilization; the more negative it is, the more stable the structure. Furthermore, it has been shown from the Helmholtz free energy *F* that the thermodynamic stability sequence is Zn_2_Zr_3_ > ZnZr > Zn_2_Zr > MgZn_2_, and Zn_2_Zr_3_ is the most thermodynamically stable compound. 

### 3.2. Elastic Constants, Polycrystalline Moduli

The calculated elastic constants of the various phases are shown in Table 2. In reality, Zn-Zr intermetallic compounds exhibit much better resistance to deformation than MgZn_2_ due to larger elastic constants; not only regarding *C*_11_ and *C*_33_ under uniaxial stress along the x or z axes, respectively, but also other compression moduli (*C*_12_ and *C*_13_) and shear moduli (*C*_44_, and *C*_66_). At this point it may be clear that ZnZr, Zn_2_Zr, and Zn_2_Zr_3_ are effective strengthening phases in Mg-Zn-Zr alloys, aside from MgZn_2_. For hexagonal MgZn_2_ and tetragonal Zn_2_Zr_3_ crystals *C*_11_ = *C*_22_ ≠ *C*_33_, the difference between *C*_11_ (*C*_22_) and *C*_33_ indicates that the two crystals have relatively strong anisotropic elastic constants, resulting in the directional dependence of the moduli. Interestingly, for hexagonal MgZn_2_ and tetragonal Zn_2_Zr_3_ crystals, the values of *C*_33_ is larger than that of *C*_11_, implying that the chemical bonds in the direction of [001] are stronger than those along the direction of [100]. The stronger chemical bonds result in hard compressing under uniaxial stress along the z axes. Moreover, the relatively large difference between *C*_11_ and *C*_33_ of MgZn_2_ implies that there is greater anisotropy on the directional dependence of the moduli than Zn_2_Zr_3_. 

**Figure 2:**
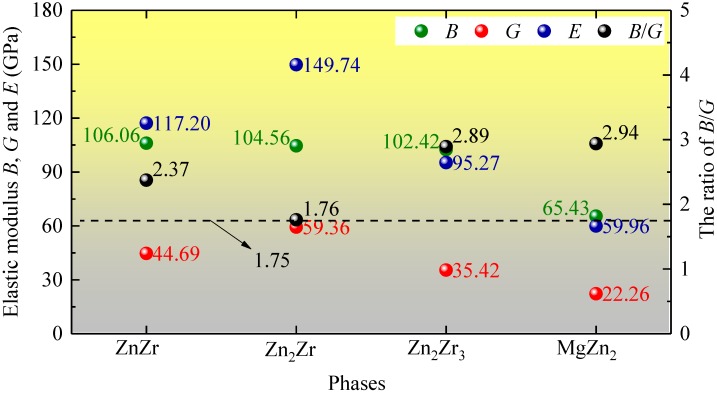
Calculated mechanical properties, including bulk modulus *B*, shear modulus *G*, and Young’s modulus *E*, as well as *B*/*G* values for ZnZr, Zn_2_Zr, Zn_2_Zr_3_, and MgZn_2_.

In order to synthetically estimate the mechanical properties, the polycrystalline bulk modulus *B*, shear modulus *G*, and Young’s modulus *E* were calculated via Voigt-Reuss-Hill approximations [30,31,32]. Figure 2 summarizes the calculated mechanical performance parameters. Notably, the *B* values of ZnZr, Zn_2_Zr, and Zn_2_Zr_3_ are close, and are all larger than that of MgZn_2_. The larger *B* values responded to the stronger capacity of the resist deformation, reflecting the good resistance of the selected Zn-Zr intermetallic compounds to deformation. Meanwhile, the *B* values of all considered intermetallic compounds are in good agreement with the fitting bulk modulus, *B*_0_. The shear modulus *G* of the system in descending order is: Zn_2_Zr > ZnZr > Zn_2_Zr_3_ > MgZn_2_. It is clear that the Young’s modulus *E* has the same order as the shear modulus *G*, suggesting that Young’s modulus *E* of the considered polycrystalline materials is more sensitive to the shear modulus than the bulk modulus. Relatively larger mechanical parameters, including bulk modulus *B*, shear modulus *G*, and Young’s modulus *E*, prove that Zn_2_Zr has outstanding mechanical properties and pronounced strengthening effects among all strengthening phases. In contrast, the lowest *B*/*G* value 1.76 reveals its brittle characteristics relative to other phases, although the material behaves as ductile when the B/G ratio >1.75 [33]. Analysis of Figure 2 thus allows us to conclude that ZnZr and Zn_2_Zr_3_ serve to combine the natures of high strength and great ductility.

### 3.3. Electronic Structures

**Figure 3:**
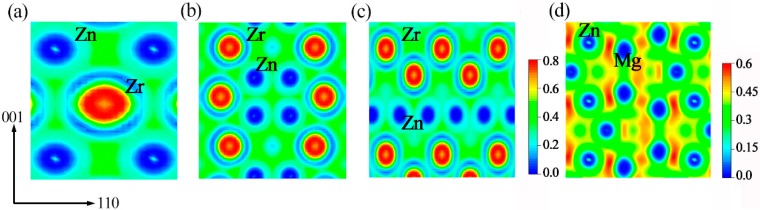
The contour line diagrams of the electron localization function (ELF) electronic distributions for Zn-Zr and MgZn_2_ intermetallic compounds on the (110) plane—namely, ZnZr (**a**), Zn_2_Zr (**b**), Zn_2_Zr_3_ (**c**), and MgZn_2_ (**d**). The interval between the two nearest contour lines is 0.2 and 0.15 for the two systems, respectively.

To gain further insight into the reasons for the Zn-Zr system strengthening, the electron localization function (ELF) [34] was applied to assist in identifying the distribution of the charges and the bonding condition. As a general rule, the ELF value is on the range 0 ≤ ELF ≤ 1, where ELF = 0, 1 corresponds to the completely delocalized state and the perfect localization, respectively. The electron localization functions on the (110) plane for all selected intermetallic compounds are presented in Figure 3. Clearly, there are obvious ionic characteristics in ZnZr, Zn_2_Zr, and Zn_2_Zr_3_ intermetallic compounds due to the delocalization around Zn and localization around Zr. Meanwhile, Zn-Zn and Zr-Zr present typical metal bond characteristics because of the even distribution of charges between the component atoms. In contrast, Mg-Zn bonds show covalent characteristics based on the apparent accumulation of charge distribution between Mg and Zn atoms in MgZn_2_ alloys. This result is consistent with the investigation of Reference [35], where more hybridized peaks between Mg p and Zn p appear near the Fermi level, indicating the presence of strong covalent bonding. Based on the above discussion, the bonding characteristics in Zn-Zr and MgZn_2_ intermetallic compounds play a role in determining a ductile or brittle nature, meaning that ionic bonds in Zn-Zr intermetallic compounds cause them to have lower ductility than MgZn_2_. In addition, for Zn-Zr intermetallic compounds, the strength of the ionic bond was also compared based on the result of charge transfer using the Bader charge analysis. For a reasonable and intuitive comparison, the average charge transfer amount of per Zr atom (e/atom) in Zn-Zr intermetallic compounds could be used as a basis, and the descending order is: Zn_2_Zr (1.01) > ZnZr (0.95) > Zn_2_Zr_3_ (0.72). From the perspective of ionic bonds, the strength of the Zn_2_Zr phase is stronger than ZnZr and Zn_2_Zr_3_, which is entirely consistent with the above elastic moduli results.

### 3.4. Minimum Thermal Conductivity and Anisotropy

It is well-known that thermal conductivity is inversely proportional to temperature. At elevated temperatures, the thermal conductivity will decrease to a limit value considered as the minimum thermal conductivity, which can be developed to identify candidate materials for high-temperature applications [36,37]. For the purpose of precisely calculating the minimum thermal conductivity of selected Zn-Zr and MgZn_2_ intermetallic compounds with anisotropic chemical bonds, the modified Clarke relation by Liu et al. [16] was used as defined:

(4) $$k_{\min}\to k_{B}\nu_{m}{\left(\frac{M}{npN_{A}}\right)}^{-2/3}$$

where *k*_B_ is the Boltzmann’s constant, *v*_m_ is the average sound velocity, *N*_A_ is Avogadro’s number, *ρ* is the density, *M* is the molecular weight, and *n* is the number of atoms in the molecule. The average sound velocity *v*_m_ is given by [38,39]:

(5) $$\upsilon_{m}={\left[\frac{1}{3}\left(\frac{2}{\upsilon_{t}^{3}}+\frac{1}{\upsilon_{l}^{3}}\right)\right]}^{-1/3}$$

(6) $$\upsilon_{t}=\sqrt{\frac{G}{\rho }}$$

(7) $$\upsilon_{l}=\sqrt{\frac{B+4/3G}{\rho }}$$

where *B* and *G* are the bulk modulus and shear modulus, respectively. Using Liu’s model, the minimum thermal conductivity of ZnZr, Zn_2_Zr, Zn_2_Zr_3_, and MgZn_2_ is 0.48, 0.67, 0.68, and 0.49 (W·m^−1^·K^−1^), respectively. Since the values of the minimum thermal conductivities of the Zn-Zr intermetallic compounds such as Zn_2_Zr and Zn_2_Zr_3_ are far larger than MgZn_2_, it can be proved that the thermal conductivity of the Mg-Zn-Zr alloy will be markedly improved as the precipitation of Zn atoms from the α-Mg matrix form Zn-Zr intermetallic compounds other than MgZn_2_. Meanwhile, Zn_2_Zr_3_, with maximum thermal conductivity, can be considered as the most important contribution to the total thermal conductivity due to its minimum Helmholtz free energy, which is considered to be a key factor for the alloys to be formed. However, for ZnZr, the difference in the minimum thermal conductivity between them could still be ignored.

Furthermore, an important question to ask is how the minimum thermal conductivity along a different direction can affect the overall minimum thermal conductivity. To clarify this point, the directional dependence of the minimum thermal conductivity can be computed from the quasi-transverse or quasi-longitudinal sound velocities and the number density of atoms per mole (*n*) of the compound, according to Cahill’s model [17]:

(8) $$K_{\min}=\frac{k_{B}}{2.48}n^{2/3}\left(v_{l}+v_{t1}+v_{t2}\right)$$

for tetragonal Zn_2_Zr_3_ crystal structure symmetry, the acoustic velocities can simply be written as:

$$\left[100\right]=\left[010\right]\nu_{l}=\sqrt{C_{11}/\rho };\;\left[001\right]\nu_{t1}=\sqrt{C_{44}/\rho };\;\left[010\right]\nu_{t2}=\sqrt{C_{66}/\rho }$$

$$\left[001\right]\nu_{l}=\sqrt{C_{33}/\rho };\;\left[100\right]\nu_{t1}=\left[010\right]\nu_{t2}=\sqrt{C_{66}/\rho }$$

$$\left[110\right]\nu_{l}=\sqrt{\left(C_{11}+C_{12}+2C_{66}\right)/2\rho };\;\left[001\right]\nu_{t1}=\sqrt{C_{44}/\rho };\;\left[0\bar{1}0\right]\nu_{t2}=\sqrt{\left(C_{11}-C_{12}\right)/2\rho }$$

for hexagonal MgZn_2_:

$$\left[100\right]\nu_{l}=\sqrt{\left(C_{11}-C_{12}\right)/2\rho };\;\left[010\right]\nu_{t1}=\sqrt{C_{11}/\rho };\;\left[010\right]\nu_{t2}=\sqrt{C_{44}/\rho }$$

$$\left[001\right]\nu_{l}=\sqrt{C_{33}/\rho };\;\left[100\right]\nu_{t1}=\left[010\right]\nu_{t2}=\sqrt{C_{44}/\rho }$$

for cubic ZnZr and Zn_2_Zr:

$$\left[100\right]=\left[010\right]=\left[001\right]\nu_{l}=\sqrt{C_{11}/\rho };\;\left[010\right]\nu_{t1}=\left[010\right]\nu_{t2}=\sqrt{C_{44}/\rho }$$

$$\left[110\right]\nu_{l}=\sqrt{\left(C_{11}+C_{12}+2C_{44}\right)/2\rho };\;\left[1\bar{1}0\right]\nu_{t1}=\sqrt{\left(C_{11}-C_{12}\right)/2\rho };\;\left[001\right]\nu_{t1}=\sqrt{C_{44}/\rho }$$

**Figure 4:**
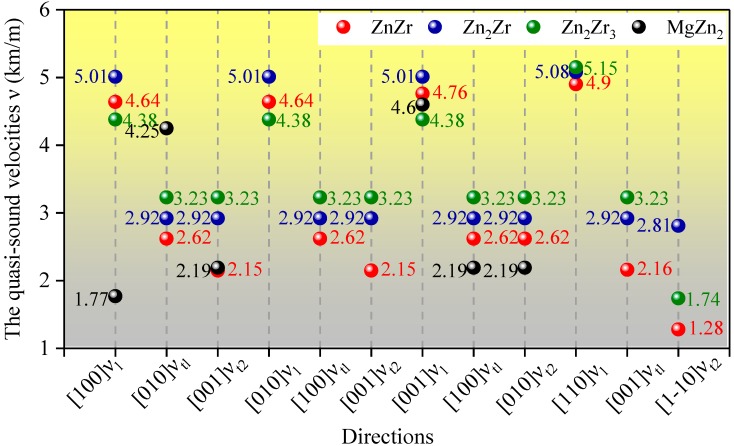
The calculated transverse and longitudinal acoustic velocities along different directions for ZnZr, Zn_2_Zr, Zn_2_Zr_3_, and MgZn_2_.

The calculated acoustic velocities along different crystal directions are shown in Figure 4. It is clear that Zn_2_Zr exhibits relatively little deviations in terms of all considered acoustic velocities due to the small variation in elastic constants. All Zn-Zr intermetallic compounds present better symmetry results along the [100], [010], and [001] directions compared with MgZn_2_, and this may be the result of its hexagonal symmetry. Interestingly, the difference between quasi-transverse and quasi-longitudinal acoustic velocities for each Zn-Zr crystal structure along the [110] direction are most pronounced, indicating that the most diverse chemical bonds are in this direction. However, the quasi-longitudinal sound velocity of all Zn-Zr intermetallic compounds in the [110] direction are closest to each other, probably because they exhibit similar ionic bonds in the [110] direction.

**Figure 5:**
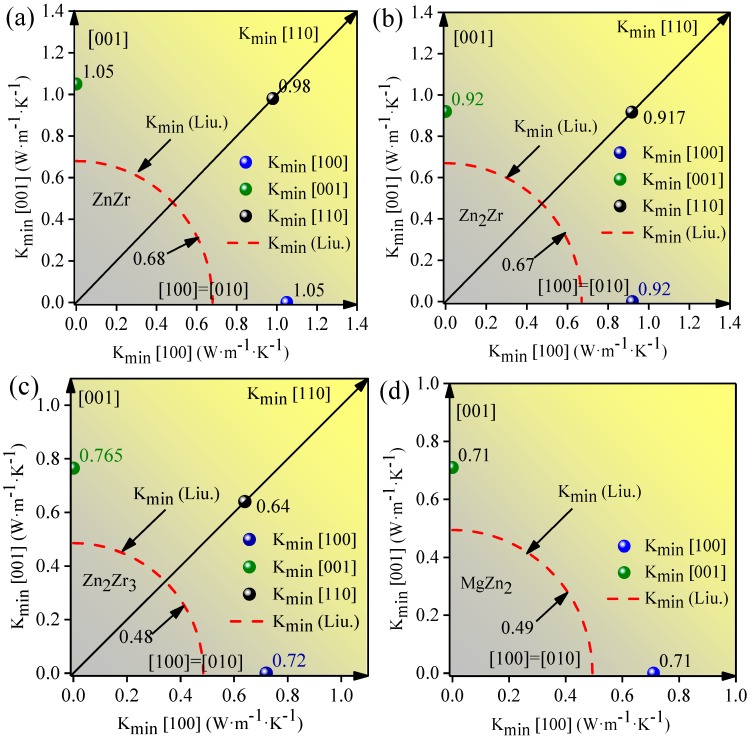
The minimum thermal conductivities along different principal directions of alloys, evaluated using Cahill’s equation and Liu’s model. (**a**) ZnZr; (**b**) Zn_2_Zr; (**c**) Zn_2_Zr_3_ and (**d**) MgZn_2_.

In addition, the theoretical minimum thermal conductivities along different principle directions can be obtained using the acoustic velocities, as shown in Figure 5. For all considered intermetallic compounds, the directional dependence of the minimum thermal conductivity obtained in the present calculation is around 30% higher than those obtained by the Liu relation. Nevertheless, by comparing the values of minimum thermal conductivity in different directions, we can still understand the anisotropy of the minimum thermal conductivity to some extent. As can be observed, the minimum thermal conductivities of the ZnZr, Zn_2_Zr, and MgZn_2_ intermetallic compounds are the same in the three principle axis directions, indicating that the limit of thermal conductivity along the *x*, *y*, and *z* axes are directionally insensitive. Compared with ZnZr, Zn_2_Zr, and MgZn_2_, the minimum thermal conductivity of Zn_2_Zr_3_ along the *z* axis is higher than the *x* and *y* axes. What is striking in this figure is the minimum thermal conductivity along the [110] direction. It can be seen that the value of minimum thermal conductivity along the [110] direction is the smallest for all considered directions, indicating that Zn-Zr ionic bonding in the [110] direction will greatly affect the thermal conductivity at high temperatures, which will result in slower heat dissipation in the [110] direction. The minimum thermal conductivity in the [110] direction will be a crucial factor when considering the minimum thermal conductivity for the selected Zn-Zr intermetallic compounds, especially for Zn_2_Zr_3_. This fact definitely indicates that the bonding anisotropy, reflected in the elastic constant anisotropy, leads to anisotropy in the high-temperature limit of thermal conductivity—that is, the minimum thermal conductivity. 

## 4. Conclusions

In summary, the structural stability, mechanical properties, bonding characteristics, and minimum thermal conductivity for the intermetallic compounds ZnZr, Zn_2_Zr, Zn_2_Zr_3_, and MgZn_2_ have been investigated by first-principles calculations. Moreover, the crystal direction/minimum thermal conductivity relationship of the materials were also established. 

Based on the difference between *C*_11_ and *C*_33_, MgZn_2_ was found to possess greater anisotropy on the directional dependence of the modulus than Zn_2_Zr_3_. ZnZr and Zn_2_Zr_3_ was found to combine the natures of high strength and great ductility. Strong ionic bonds in ZnZr, Zn_2_Zr, and Zn_2_Zr_3_ was found to lead to the characteristics of a higher modulus but lower ductility than MgZn_2_. Furthermore, the strength of the Zn_2_Zr phase was found to be stronger than ZnZr and Zn_2_Zr_3_ based on the maximum charge transfer using Bader’s charge analysis. Based on the calculated minimum thermal conductivities of the intermetallic compounds ZnZr, Zn_2_Zr, Zn_2_Zr_3_, and MgZn_2_, we conclude that the thermal conductivity of the Mg-Zn-Zr alloy will be markedly improved as the precipitation of Zn atoms from the α-Mg matrix help to form Zn-Zr binary alloys. However, the minimum thermal conductivity along the [110] direction may serve to be a crucial limit.

## Figures and Tables

**Table 1:** The calculated lattice parameters at the ground state (*a*, *c* in Å, *ρ* in g/cm^3^) and the fitting bulk modulus *B*_0_ (GPa) at the spin state, as well as the Helmholtz free energy *F* (eV/atom) at 0 K. For MgZn_2_, the *B*_0_ responds to the no-spin state.

Species	Space Group	Lattice Parameters		*ρ*	*B*_0_	*F*
		*a*	*c*			
ZnZr	*Pm* $\bar{3}$ *m*	3.34 (3.34 ^a^)	-	7.00	107.11	−5.07
Zn_2_Zr	*Fd* $\bar{3}$ *m*	7.39 (7.40 ^a^)	-	7.31	104.47	−3.83
Zn_2_Zr_3_	*P*4_2_/*mnm*	7.59 (7.63 ^a^)	6.83 (6.76 ^a^)	6.84	102.54	−5.71
MgZn_2_	*P*6_3_/*mmc*	5.23 (5.20 ^b^)	8.56 (8.54 ^b^)	5.17	63.69	−1.34

^a^ From Reference [8]. ^b^ From Reference [26].

**Table 2:** The calculated independent elastic constants (GPa) of Zn-Zr intermetallic compounds and MgZn_2_ using the strain-energy method with other calculated (Cal.) and experimental (Exp.) data.

Species	Reference	*C*_ij_					
		*C*_11_	*C*_12_	*C*_13_	*C*_33_	*C*_44_	*C*_66_
ZnZr	This work	134.04	91.82	-	-	72.96	-
	Cal. [27]	141.00	91.00	-	-	71.00	-
Zn_2_Zr	This work	183.72	68.59	-	-	62.36	-
Zn_2_Zr_3_	This work	145.94	90.00	73.72	155.22	31.76	47.00
MgZn_2_	This work	93.30	60.88	32.75	109.53	24.76	-
	Cal. [28]	91.25	87.27	28.62	147.59	20.21	-
	Exp. [29]	107.25	45.45	27.43	126.40	27.70	--
